# Cystatin C, Vitamin D and Thyroid Function Test Profile in Chronic Kidney Disease Patients

**DOI:** 10.3390/diseases9010005

**Published:** 2021-01-03

**Authors:** Marlene Tapper, Donovan A. McGrowder, Lowell Dilworth, Adedamola Soyibo

**Affiliations:** 1 Department of Pathology, Faculty of Medical Sciences, The University of the West Indies, Kingston 7, Jamaica; teeanmarl@gmail.com (M.T.); lowell.dilworth02@uwimona.edu.jm (L.D.); 2 Department of Medicine, Faculty of Medical Sciences, The University of the West Indies, Kingston 7, Jamaica; adedamola.soyibo02@uwimona.edu.jm

**Keywords:** kidney, disease, thyroid, vitamin D, cystatin C, prevalence, deficiency, insufficiency, chronic

## Abstract

Background: The progression of chronic kidney disease (CKD) is concomitant with complications, including thyroid dysfunction, dyslipidemia and cardiovascular diseases. The aim of this study is to determine serum cystatin C levels, and the prevalence of vitamin D deficiency and thyroid dysfunction in CKD patients. Methods: A cross-sectional study was conducted involving 140 CKD patients (stages 1–5) that were referred to a renal clinic. Demographic data was collected and thyroid function tests, serum 25-OH-vitamin D, cystatin C levels, and routine biochemistry tests were determined using cobas 6000 analyzer. Results: 129 (92.1%) of CKD patients had elevated serum cystatin C levels and there was a stepwise increase from stage 1–5. Overt hypothyroidism was present in one patient and nine had subclinical hypothyroidism. There was a stepwise reduction in serum 25-OH-vitamin D levels from stage 2–5, 31 (22.1%) had vitamin D insufficiency and 31 (22.1%) presented with deficiency. Conclusions: 25-OH-vitamin D deficiency and thyroid disorders are exhibited in chronic kidney disease patients and the severity of the former rises with disease progression, as indicated by elevated cystatin C levels. Routine screening and timely intervention is recommended so as to reduce the risk of cardiovascular diseases.

## 1. Introduction

Globally, chronic kidney disease (CKD) is recognized as an important public health concern as it significantly contributes to health care expenses, morbidity as well as mortality from non-communicable disease [1]. Chronic kidney disease is defined as the existence of kidney impairment and diminished function [e.g., glomerular filtration rate (GFR) less than <60 mL/min/1.73 m^2^ or albumin excretion rate ≥ 30 mg/24 h] lasting greater than three months [2]. Defining and staging chronic renal failure is contingent on assessing the GFR, and an estimate is provided by creatinine clearance. Serum creatinine, which is used to determine the creatinine clearance, is not a very sensitive indicator of renal disease and there are a number of limitations associated with obtaining an accurate measurement of the urine creatinine concentration [3].

A practical approach in determining the GFR in clinical practice is the estimated glomerular filtration rate (eGFR) that is determined by utilizing equations and which provide a more accurate and precise assessment of renal function [4]. These formula-based equations such as Modification of Diet in Renal Disease (MDRD) and Cockcroft-Gault are employed in the determination of eGFR and utilized patient-related parameters such as gender, age, body weight, ethnicity and serum creatinine concentrations [5]. These GFR-based formulas, as well as the CKD-EPI equation, are endorsed by regulatory agencies and there are clinical practice guiding principles for the routine assessment of GFR. Measured GFR utilizing endogenous or exogenous markers is commended as a confirmatory test when more accurate evaluation is necessary [6].

Cystatin C, an endogenous marker for measuring GFR is a 13 kD non-glycosylated simple protein that is synthesized and secreted by all nucleated cells at a continual rate. This endogenous serum biological marker possesses a cationic nature and thus is freely filtered via the glomerulus [7]. It possesses other characteristics that are present in steady concentrations in the plasma, not secreted by tubular cells, and compared with creatinine, it is less affected by non-renal factor influences such as gender, age, muscle mass and analytical interfering substance during its measurement [8]. Studies have demonstrated that serum cystatin C is a sensitive biomarker for detecting changes in GFR and identifying preclinical renal disease, particularly in diabetic patients with a normal serum creatinine concentration [9]. Furthermore, cystatin C is a better discriminator between type 2 diabetic patients with normoalbuminuria or microalbuminuria and its quantification is useful in the early identification of diabetic nephropathy allowing for the appropriate intervention and management [10].

There is increasing data that the synthesis of cystatin C could be activated by thyroid hormones and it may readily respond to minor alterations in the function of the thyroid glands [11,12]. The kidneys play a vital role in the metabolism and elimination of thyroid hormones. In chronic renal disease, there is significant reduction in kidney function accompanied by iodine retention, metabolic acidosis and alteration of thyroid function [13]. There are a number of observational studies that have reported various modifications of thyroid function test profile in chronic kidney disease patients [14,15]. Thyroid dysfunction ensues in chronic kidney disease with a significant decrease in total and free T4, total and free T3 and elevated TSH that is associated with increasing renal impairment and reduced GFR [14,16,17]. In chronic kidney disease patients that present with overt or subclinical hypothyroidism, there was a positive correlation between serum urea and creatinine concentrations with TSH, and negative correlation between these renal function biomarkers and FT4 and FT3 [14,16,17]. However, there are studies that have reported unchanged TSH [18,19] as well as normal TSH and FT4 [20] in chronic kidney disease patients compared to controls. Notwithstanding these studies, thyroid status in patients with chronic renal disease remains inconclusive.

The thyroid gland may be affected via immune-mediated developments as well as vitamin D impeding the dose-dependent uptake of iodide stimulated by thyroid stimulating hormone [21]. Vitamin D is a fat-soluble steroid prohormone with a cytosolic receptor produced from 7-dehydrocholesterol in the skin following sunlight exposure with the subsequent synthesis of vitamin D3 [22]. Vitamin D3 is transported to liver where metabolic activation involves hydroxylation at position 25 to form 25-hydroxyvitamin D, which is consequently converted to the biologically active 1,25 dihydroxyvitamin D by the catalytic activity of 1α-hydroxylase present in the kidney’s proximal tubular epithelial cells [23]. Vitamin D insufficiency or deficiency is very prevalent amongst chronic kidney disease patients and there appears to be an inverse association between serum concentrations of 25-hydroxyvitamin D and renal function [24]. The causes of vitamin D deficiency is due to a number of factors including: (i) increased fibroblast growth factor 23 (FGF23) levels that decrease the expression and activity of 1α-hydroxylase and stimulate 24-hydroxylase that degrades 1,25 dihydroxyvitamin D [25] (ii) increase phosphate retention that inhibit the activity of 1α-hydroxylase (iii) reduced renal mass and less proximal tubular cells with 1α-hydroxylase activity and (iv) renal loss of loss of 25-hydroxyvitamin D and its binding, particularly in chronic kidney disease patients with proteinuria [26].

Circulating 25-hydroxyvitamin D levels is regarded as a sensitive determination of vitamin D status [27] and the prevalence of vitamin D insufficiency and deficiency was found to be higher in type 2 diabetic patients with chronic kidney disease and albuminuria compared to those without albuminuria [28]. In a recent study, lower 25-hydroxyvitamin D concentration was associated with reduced GFR in type 2 diabetic patients, but there was no difference between patients with elevated urinary albumin excretion compared to those with normoalbuminuria [29].

This study aimed to determine and compare the levels of serum cystatin C in patients with chronic kidney disease (stages 1–5) based on creatinine clearance values and association with serum creatinine. This study is also aimed at determining the prevalence of vitamin D deficiency or insufficiency and thyroid dysfunction in these patients.

## 2. Materials and Methods

### 2.1. Patient Population and Ethical Approval

Patients from across Jamaica, and sometimes other Caribbean islands, were referred to the renal clinic at the University Hospital of the West Indies (UHWI) for management of CKD. This was a cross-sectional study conducted between February 2016 and May 2016. All patients attending the renal clinic were approached. After explaining the aims of the study, patients who agreed to participate were recruited. They were assigned data entry numbers in order to maintain confidentiality.

We received approval from the University of the West Indies/University Hospital of the West Indies Faculty of Medical Sciences Ethics Committee and the protocol for the conduct of research outlined were followed. Informed consent was obtained after which the patients were given directives on the proper collection of a 24 h urine sample. They were asked to repeat the instructions to ensure comprehension.

Fisher’s z test was used to estimate the sample size for a one-sample correlation test. The estimated sample size was 259 (using alpha = 0.05, power = 0.9, delta = 0.2, r° = 0 and ra = 0.2). Between February and May 2016, 140 patients from 18 to 97 years of age were recruited. The study included cases from all Jamaica encompassing those from western parishes, St. James, Hanover, Westmoreland and St. Elizabeth, who were more likely to visit the Cornwall Regional Hospital in St. James due to proximity.

### 2.2. Demographic Data Collection

Demographic data, including age, gender, date of birth, height, weight, marital status, education level and employment status were collected. Height and weight were measured in the renal clinic. The date of CKD diagnosis, age, stage and cause of renal impairment at diagnosis as well as any comorbid conditions were ascertained from the patients themselves and/or confirmed by medical record search.

### 2.3. Sample Collection and Preparation

Samples were processed within three hours of receipt in the Chemical Pathology Laboratory at the Department of Pathology, The University of the West Indies. Specimens were stored at −70 °C for assays not completed within 24 h. Serum and urine biochemistry tests with the exception of urine protein was performed on the cobas 6000 (Roche/Hitachi, Roche Diagnostics, Indianapolis, IN, USA) analyser.

### 2.4. Serum Assays Used to Determine Analytes

Seven (7) mL of blood was obtained from the patient by venipuncture from the antecubital fossa or another convenient site. Samples were obtained using a vacutainer system. Samples were allowed to clot for 30 min, separated by centrifugation at 3500 rpm for 5 min then the serum was aliquoted.

Analytes measured were serum creatinine, urea, electrolytes, albumin, uric acid, calcium, phosphorus, cystatin C, 25-hydroxyvitamin D [25-(OH)D], free thyroxine (FT4) and thyroid stimulating hormone (TSH).

An IDMS traceable Jaffé method, a kinetic colorimetric assay, was used to measure serum creatinine [30]. Cystatin C was assayed by a particle enhanced immunoturbidimetric assay, the Tina-quant Cystatin C Gen. 2, which is standardized against ERM-DA471/IFCC (The International Federation for Clinical Chemistry and Laboratory Medicine) reference material. Anti-cystatin C-coated latex particles bind cystatin C in the specimen. The degree of turbidity at 546 nm is equivalent to the cystatin C concentration [31].

Albumin was determined based on its binding to bromcresol green at pH 4.1 with the formation of a blue-green complex. The color intensity photometrically measured at 570 nm is equivalent to the level of albumin in the serum [32]. The serum calcium was determined due to the reaction of calcium ions with 5-nitro-5’-methyl-BAPTA (NM-BAPTA) under alkaline conditions to form a complex, which is further complexed with ethylenediaminetetraacetic acid (EDTA) in a second reaction. The levels of calcium are determined by the difference in absorbance at 340 nm [33].

The serum uric acid was determined as the analyte is cleaved by uricase to form allantoin and hydrogen peroxide. Peroxidase and *N*-ethyl-*N*-(2-hydroxy-3-sulfopropyl)-3-methylaniline (TOOS) catalyze the oxidation of 4-aminophenazone to produce a dye (quinone-diimine). The color intensity of the dye is proportionate to the uric acid concentration. The increasing absorbance at 546 nm is measured [34].

Free thyroxine (FT4) was measured by an electrochemiluminescence immunoassay (ECLIA) (Roche Diagnostics, Indianapolis, IN, USA), which uses a specific anti-T4 antibody labelled with a ruthenium complex. A competitive method is employed and the concentration of T4 is inversely proportional to the chemiluminescent emission [35]. Thyroid stimulating hormone (TSH) was measured by an assay that employs a sandwich principle. Two monoclonal antibodies specific for TSH, one biotin-labelled and the other ruthenium-labelled react with TSH in serum to form a sandwich complex. Streptavidin-coated micro-particles are added and binds biotin. Microparticles are bound magnetically to an electrode and a reaction is carried out. The concentration of TSH is proportional to the chemiluminescent emission [36].

Total 25-hydroxyvitamin D [25-(OH)D] was measured by a competitive immunoassay that utilizes a vitamin D binding protein as a capture protein. Pretreatment reagents release hydroxylated vitamin D from vitamin D binding protein. With the addition of a ruthenium-labelled VDBP (VDBPr), a complex is formed with the hydroxylated vitamin D. Microparticles coated with streptavidin and biotin-labelled 25-(OH)D [25-(OH)Db] are added and attach to unbound VDBPr. A complex is formed. The micro-particles are bound electromagnetically and unbound substances are removed by washing. Chemiluminescence is induced by the application of a voltage. The emission is quantified by a photomultiplier and is inversely proportional to the concentration of 25-(OH)D [37].

Patients were placed into the five stages of CKD determined by 24 h creatinine clearance (CrCl).

### 2.5. Statistical Analysis and Predictor Levels of Cystatin C, Vitamin D and Thyroid Status

The data analysis was conducted using the IBM Statistical Programme of the Social Science (SPSS) version 22 and Microsoft Excel. Demographic characteristics are presented as mean ± standard deviation (SD). The frequencies of different causes of CKD were determined. The Pearson coefficient (r) was used to assess the correlation of results by serum cystatin C and CrCl.

Cystatin C levels were divided into normal and abnormal based on the quoted normal range of 0.61–0.95 mg/L and compared to serum creatinine using the independent *t*-test. Vitamin D levels were (i) deficient < 20 ng/mL, (ii) insufficient 20–29 ng/mL and (iii) sufficient ≥ 30 ng/mL. Mean values according to gender and stages of kidney disease were ascertained. The association of hypovitaminosis D with diabetes mellitus was determined by the independent *t*-test. A *p*-value < 0.05 (two-tailed) indicated statistical significance.

The prevalence of thyroid disorders in the subjects was assessed. Patients were classified as (i) overt hyperthyroidism (TSH < 0.4 mU/L and FT4 > 1.9 ng/mL), (ii) sub-clinical hyperthyroidism (TSH < 0.4 mU/L and FT4 normal), (iii) overt hypothyroidism (TSH > 4.0 mU/L and FT4 < 0.8 ng/mL), and (iv) sub-clinical hypothyroidism (TSH > 4.0 mU/L and FT4 normal). The mean values of FT4 and TSH according to gender as well as the mean values according to CrCl compared. A *p*-value < 0.05 (two-tailed) indicated statistical significance.

## 3. Results

### 3.1. Demographic Characteristics and Aetiologies of CKD

The demographic characteristics of the subjects are given in Table 1. It was observed that 75 (53.6%) were females and 65 (46.4%) be present males. Ages of the subjects ranged from 18–98 years. The mean age of females was 55.61 ± 17.98 years while the mean age of males was 61.11 ± 17.39 years (Table 1). The age at diagnosis of CKD ranged from 7–97 years and the mean age of females at diagnosis was 50.37 ± 17.89 years and males 55.91 ± 17.32 years. The majority of the patients (27.9%) were in the 70 and over age group, had secondary level education (42.9%) and were unemployed (60.7%). There were significant differences (*p* < 0.05) between males and females in height and body surface area (BSA) (Table 1).

The aetiologies of CKD were diabetes mellitus 43 (30.7%), hypertension 26 (18.6%), HbSS, 19 (13.6%), systemic lupus erythematosus (SLE) 13 (9.3%), obstructive uropathy 13 (9.3%), chronic glomerular nephritis (CGN), 7 (5%), autosomal dominant polycystic kidney disease (ADPKD) 5 (3.6%), human immunodeficiency virus (HIV), 3 (2.1%), and unknown 1 (0.7%). Twenty-one (15%) people had other causes that were either the sole cause or in conjunction with one of the previous aetiologies. These included amyloidosis in one case, eight with solitary kidney (six post nephrectomy for renal cell carcinoma and two congenital), one patient with an ectopic horseshoe kidney and one with nephrocalcinosis. One patient had HbSC. The main cause in females was diabetes mellitus followed by SLE and hypertension/HbSS. The most prevalent cause in males was diabetes mellitus followed by hypertension and obstructive uropathy.

### 3.2. Results for Serum Cystatin C

There was no significant difference in serum cystatin C between males and female (Table 1). There were significant positive correlations between serum cystatin C and creatinine (r = 0.838, *p* < 0.05), urea (r = 0.782, *p* < 0.05) and significant negative correlation with CrCl (r = −0.623, *p* < 0.05).

Serum cystatin C levels in the 140 chronic kidney disease patients ranged from 0.68 to 8.13 mg/L (normal reference interval 0.61–0.95 mg/L). The mean serum cystatin C level was 2.32 ± 1.33 mg/L. There were 11 (7.9%) subjects with normal serum cystatin C levels (mean 0.84 ± 0.09 mg/L) while 129 (92.1%) had elevated levels (mean 2.45 ± 1.31 mg/L) (Table 2). Of the 53 subjects with normal serum creatinine levels, 42 (79.2%) had high serum cystatin C levels while none of the 87 subjects with elevated serum creatinine had normal serum cystatin C levels. Ten of the 11 patients with normal serum cystatin C levels had stage 1 CKD while one had stage 2 disease. The highest serum cystatin C levels (4.46 ± 1.34 mg/L) were seen in patients with end stage renal disease (ESRD) (Table 3). It is also noted that there was a stepwise increase in serum cystatin C from stage 1–5 (Table 3).

The independent *t*-test demonstrated significant differences in serum creatinine and creatinine clearance (CrCl) levels with normal and abnormal serum cystatin C levels (Table 2). Serum cystatin C levels were significantly higher (*p* < 0.05, two-tailed) in patients with diabetes mellitus (*n* = 43, mean 2.80 ± 1.28 mg/L) (Table 4) with levels ranging from 1.18–5.57 mg/L. Serum cystatin C levels were significantly lower in patients with SLE (*n* = 13, mean 1.50 ± 1.10 mg/L vs. 2.41 ± 1.32 mg/L, *p* < 0.05, two-tailed).

Diabetic patients with chronic kidney disease had significantly higher serum creatinine, cystatin C and urine protein levels, and 24-h CrCl. In the chronic kidney disease population, diabetic patients were significantly older than those without diabetes mellitus (Table 4).

### 3.3. Results for Thyroid Function Tests

The results for Free T4 (FT4) ranged from 0.63–2.86 ng/mL, mean 1.12 ± 0.27 ng/mL, and TSH levels ranged from < 0.01–18.3 mIU/L, mean 2.19 ± 1.95 mIU/L. Overt hyperthyroidism was observed in two (1.0%) chronic kidney disease patients while six (4.0%) subjects had subclinical hyperthyroidism. One (0.7%) chronic kidney disease patient had overt hypothyroidism and nine had subclinical hypothyroidism. Ten patients had FT4 < 0.8 ng/mL and normal TSH, the sick euthyroid syndrome also seen in chronic kidney disease patients. One patient on peritoneal dialysis and two on hemodialysis had normal thyroid hormone levels, while one hemodialysis patient had overt hypothyroidism (FT4 0.71 ng/mL, TSH 9.59 mIU/L).

There was no significant difference for FT4 and TSH at different stages of CKD (Table 3); however, a significant difference in FT4 levels was noted between males (mean 1.18 ± 0.27) and females (mean 1.07 ± 0.27) (*p* < 0.05). The patient with overt hypothyroidism had stage 5 CKD. Three persons with subclinical hypothyroidism had stage 2 while 6 had stage 3 CKD. Of the six patients with subclinical hyperthyroidism, three had stage 1, two had stage 2 and one had stage 4 CKD.

The TSH levels were highest in chronic kidney disease patients in stage 3 and the FT4 levels were lowest in stage 5 (Table 3). There was no difference in TSH or FT4 levels between patients who are diabetic compared to non-diabetic patients (Table 4).

### 3.4. Results for Vitamin D

There was a stepwise decrease in serum 25-(OH)D levels from stage 2 to 5 chronic kidney disease. Serum 25-(OH)D levels were the lowest in patients with stage 5 chronic kidney disease with mean levels < 30 ng/mL (Table 3).

While mean serum 25-(OH)D levels was normal in diabetic patients with chronic kidney disease, they were significantly lower (*p* < 0.05) than those of non-diabetic subjects (Table 4). The mean serum 25-(OH)D levels in diabetic patients was 30.16 ± 14.84 ng/mL (*n* = 43) while non-diabetic patients had mean levels of 36.05 ± 15.49 ng/mL (*n* = 97) (Table 4). The 25-(OH)D levels in patients with SLE were assessed. The prevalence of SLE in the study population was 9% (13/140). 25-(OH)D levels < 30 ng/mL was observed in 46% (*n* = 6) of subjects; five (38%) had insufficient levels, while one (7%) was deficient.

The serum 25-(OH)D levels in the study population ranged from 4–71 ng/mL with mean 34.24 ± 15.48 ng/mL. The prevalence of vitamin D insufficiency was 22.1% with mean level 25.87 ± 2.11 ng/mL, and vitamin D deficiency 22.1% with mean level 14.81 ± 4.59 ng/mL. Subjects were grouped based on sufficient versus not sufficient 25-(OH)D levels and using the independent t-test, significant differences were observed in calcium, phosphorus, albumin and urine protein levels (Table 5).

## 4. Discussion

### 4.1. Findings of Cystatin C

The mean serum cystatin C levels from stage 1 to 5 were greater than 1.10 mg/L in this group of chronic kidney disease patients. In epidemiologic studies, a raised serum cystatin C level (greater than 1.0 mg/L) in individuals with eGFR_creat_ greater than 60 mL/min per 1.73 m^2^ has been utilized to detect preclinical kidney dysfunction that is not identified by estimated GFR or serum creatinine levels [7]. There are meta-analyses that have determined that the diagnostic accuracy of serum cystatin C is undoubtedly superior to serum creatinine for identifying impaired kidney function [38,39]. In the meta-analysis by Roos et al. involving 24 studies, cut-off values of serum cystatin C, 0.9–1.4 mg/L was used to detect early renal impairment [39] and this range covers the chronic kidney disease patients in stages 1 and 2 in this study. These findings were corroborated in two studies involving type 1 diabetic patients as serum cystatin C levels gave more accurate measurements of GFR estimation compared to serum creatinine in early renal impairment [40,41].

There was a stepwise increase in mean serum cystatin C levels in this group of chronic kidney disease patients. In a large cohort of patients with type 1 diabetes, serum cystatin C levels was more sensitive for identifying early decline in kidney function and better associated with glomerular filtration rate than serum creatinine levels and creatinine-based estimated GFR formulae [42]. In addition, there was a significant stepwise elevation of serum cystatin C levels as GFR deteriorated indicating very prompt detection of decreased kidney function [42]. This finding is similar and corroborates with that of our study.

Serum cystatin C levels in stage 3–5 was greater than 2.0 mg/L and highest in stage 5. Serum creatinine was within the normal range in stage 1 and 2 but increased from mild to moderate in stages 3 and 4 and markedly in stage 5. Serum cystatin C levels were significantly higher in patients with diabetes mellitus than in their non-diabetic counterparts. Christensson et al. found that serum cystatin C is more precise than serum creatinine in approximating GFR in type 2 diabetic patients with mild to moderate chronic renal failure [43]. This finding was substantiated in a later study where serum cystatin C had a greater diagnostic accuracy in detecting impaired renal function in stage 2 and 3 chronic kidney disease patients [44]. Likewise, serum cystatin levels in 825 non-diabetic adults with stages 3 and 4 chronic kidney disease was highly predictive of end stage kidney disease and other outcomes such as cardiovascular-related mortality and all-cause mortality [45].

In this study, approximately two-fifths (37.9%) of the chronic kidney disease patients had normal serum creatinine levels and approximately four-fifths of these patients had elevated serum cystatin C. Moreover, none of the patients with elevated serum creatinine had normal cystatin C levels. Krolewski et al. reported that serum cystatin C seems to better risk stratify and a superior predictor of end renal stage disease in diabetic patients [46]. In a later study, baseline serum cystatin C level was a better predictor of end stage renal disease than measured glomerular filtration rate (mGFR) and serum creatinine for predicting end-stage renal disease in type 2 diabetic patients with raised albuminuria [47]. Moreover, a study by Christensson et al. of a large cohort of type 1 and type 2 patients with diabetes mellitus found that serum cystatin C was more operative than serum creatinine in identifying patients with early diabetic nephropathy [42]. Serum creatinine recognized 12% and serum cystatin C identified 40% of type 2 diabetic patients with nephropathy [48], while in another study, serum cystatin C discriminated between normoalbuminuria and microalbuminuria in type 2 diabetic patients [49]. In this study, the urinary albumin excretion rate in the end stage renal disease patients was not determined. Future studies could focus on examining the utility of serum creatinine, cystatin C and albuminuria.

### 4.2. Findings of Vitamin D

A key finding in this study was a stepwise decrease in serum 25-(OH)D levels from stage 2 to 5. Serum 25-(OH)D levels were lowest in persons with stage 5 chronic kidney disease with mean levels in patients with stage 5 disease less than 30 ng/mL. In this context, Lewin et al. conducted an outpatient cohort cross-sectional study performed in one hundred and fifty three centres and found low levels of 25-(OH)D defined as <22 pg/mL in over 30% of chronic kidney disease patient with stage 3 disease and vitamin D deficiency prevalence of 60%–70% in stages 4 and 5 [50]. Similarly, the prevalence of low vitamin D levels in chronic kidney disease gradually increases with the advancing severity of the disease. The prevalence of hypovitaminosis D greater than 30% and 70% was found in chronic kidney disease patients with stage 3 and stage 5 respectively [51]. Additionally, in agreement with these findings is a retrospective cross-sectional study of 152 pre-dialysis chronic kidney patients of stages 3 to 5 where serum 25-(OH)D levels of patients in stages 4 and 5 were significantly lower than that of their counterparts in stage 3 [52]. Interestingly, reduction in GFR in chronic kidney disease have been found to be proportionate to the progressive decrease in calcitriol levels, possibly due to reduced renal mass and number of tubular cells with 1α-hydroxylase activity [53]. The progressive decrease in 25-(OH)D levels from stage 1 to 5 in this study could also be due to the increase of fibroblast growth factor 23 and phosphate retention as GFR decline, resulting in suppressed expression and activity of renal 1α-hydroxylase [54].

There are organizations that have recommended varying levels of vitamin D insufficiency and deficiency and the Endocrine Society endorses 25-(OH)D levels < 20 ng/mL be designated vitamin D deficiency, 20 to 29 ng/mL levels considered vitamin D insufficiency, while levels > 30 ng/mL deemed normal [55]. These levels of 25-(OH)D are applied in different studies to patients with chronic kidney disease [56,57]. In the present study, the prevalence of vitamin D insufficiency (20–29 ng/mL) was 22.1% and also 22.1% for vitamin D deficiency (≤20 ng/mL) in the chronic kidney disease patients. There are numerous studies that have established that persons with chronic kidney failure are at risk of vitamin D deficiency [58,59,60,61]. In a prospective cross-sectional study involving 50 chronic kidney disease stages 2 to 4 by Rozita et al., the prevalence of vitamin D insufficiency (15–30 ng/mL) was 60.0% and vitamin D deficiency (<15 ng/mL) was 40.0% [62].

In a single center observation study of 43 pre-dialysis chronic kidney disease patients (stages 1–5) and 103 patients undergoing hemodialysis, inadequate levels of 25-(OH)D (<30 ng/mL) was found in 86.0% of the former and 97% of the latter with no correlation of 25-(OH)D levels with either albumin or parathyroid hormone levels [58]. Furthermore, the prevalence of vitamin D deficiency (<15 ng/mL) in patients with chronic kidney disease patients with stage 5 undergoing hemodialysis was 22.6% and vitamin D insufficiency (15–30 mg/mL) 53.5% [59]. In addition, in a prospective multicenter cohort study of 201 chronic disease patients from 12 geographically diverse regions of the United States, only 29% of individuals with stage 3 and 17% with stage 5 had sufficient levels of 25-(OH)D [61]. All these studies are in agreement that there are significant sub-optimal levels of vitamin D in chronic kidney disease patients. The differences in prevalence of vitamin D deficiency or insufficiency may be due to the stages of the chronic renal disease patient population and the levels of vitamin D that defines insufficiency or deficiency amongst other factors. Other contributing factors to inadequate levels of 25-(OH)D are proteinuria, nutritional factors and inadequate sunlight exposure [63,64,65]. The lower prevalence of vitamin D insufficiency and deficiency of the chronic kidney disease patients in our study compared to others may be due to the patients having adequate exposure to sunlight in Jamaica throughout the year.

In this study, the prevalence of SLE in the study population was 9.0% and of this amount, vitamin D levels < 30 ng/mL was observed in 46% of subjects. A number of studies have assessed the prevalence of vitamin D insufficiency and deficiency in patients with SLE [66,67]. Elsaid et al. conducted a cross-sectional study involving the evaluation of vitamin D levels in SLE patients and health controls and found vitamin D insufficiency (15–30 ng/mL) in 65% and vitamin D deficiency (<15 ng/mL) in 23.3% of patients [68]. In another cross-sectional study involving 90 SLE patients, 75.0% of patients of patients presented with vitamin D insufficiency (<30 ng/mL) and vitamin D deficiency (<10 ng/mL) [69]. However, there are not many studies that have assessed an association among SLE patients with renal disease and inadequate levels of vitamin D. In a population-based cohort of 123 SLE patients, 67.0% of individuals presented with vitamin D deficiency and 17.9% had critically low levels (<10 ng/mL) with the incidence of renal disease being the most important predictor followed photosensitivity [70].

Diabetes mellitus was the main cause of chronic kidney disease in our study population and the mean levels of 25-(OH)D in these patients, although in the normal range, it was significantly lower than that of non-diabetic subjects. Diabetic patients had significantly higher creatinine, cystatin C and urine protein concentrations, and significantly lower 24-h CrCl. Many studies have established that diabetic patients with chronic kidney disease are at risk of presenting with less than optimal vitamin D levels [71,72,73,74]. In agreement with our findings is a report by Wahl et al. that examined the data from the Chronic Renal Insufficiency Cohort Study. Individuals diagnosed with diabetes mellitus and chronic renal disease stages 2 to 4 presented with significantly lower vitamin D levels compared to non-diabetics [74]. Likewise, in 2015, Peng et al. found insufficient levels (median value of 8.5 ng/mL) in 93.1% of 144 patients with type 2 diabetes mellitus and chronic kidney disease compared with 78.9% of 304 non-diabetic patients with chronic kidney disease [72]. Moreover, in a retrospective study executed by Sipahi et al. that registered 1463 patients with type 2 diabetes mellitus and chronic kidney disease, 52.0% of the subjects had vitamin D deficiency and 24.0% presented with vitamin insufficiency [73]. However, a study by Yaturus et al. showed insufficient levels of vitamin D levels in both diabetic and non-diabetic patients with chronic renal failure and no-significant difference between the two groups [75].

### 4.3. Findings of Thyroid Function Tests

In this study, we observed a reducing trend for FT4 levels, although it was not significant from stage 3 to 5, and an increasing trend for TSH across chronic kidney disease across stages 1 to 3, and stages 4 and 5. The TSH levels were highest in chronic kidney disease patients in stage 3 and the FT4 levels were lowest in stage 5. Previous studies are in agreement with our findings [18,76,77]. Khatiwada et al. found a non-significant decreasing trend for FT4 and FT3, but a significant rise for TSH levels across stages 3 to 5 in 360 chronic kidney disease [78]. There was a parallel increase in hypothyroidism with the progression of chronic kidney disease with prevalence of subclinical hypothyroidism 15.2% in stage 3, 32.0% in stage 4 and 43.3% in stage 5 disease [78]. Khatiwada et al. also found a non-significant decreasing trend for FT4 and FT3 but significant rise for TSH levels across stages 3 to 5 in 360 chronic kidney disease [78]. In an earlier study Singh et al. found reduced levels of FT3, FT4, albumin and total protein levels in un-dialyzed chronic renal failure patients compared to controls [79]. Likewise, in a cross-sectional study of 45 adult chronic renal disease patients and 45 sex- and age-matched controls there was significant reduction in serum total T4, total T3, albumin and total protein levels with an associated increase in TSH levels compared to healthy controls [80]. However, in a study by Rajagopalan et al. of 60 patients with 60 un-dialyzed chronic kidney disease patients, both FT4 and FT3 were significantly elevated with unaffected TSH compared to healthy controls [81].

This study revealed that there was one (0.7%) patient that had overt hypothyroidism, and nine (6.4%) had subclinical hypothyroidism. The patient with overt hypothyroidism had stage 5 chronic kidney disease, three persons with subclinical hypothyroidism had stage 2 disease, while six had stage 3 chronic kidney disease. This indicates that TSH levels generally increase with the progression of kidney impairment. We found lower prevalence of thyroid dysfunction in chronic kidney disease than reported by other observational studies. Lo et al. analyzed data from the Third National Health and Nutrition Examination Survey and establish that the prevalence of hypothyroidism in 14,623 chronic kidney patients was 23.1% for subjects with glomerular filtration rate less than 30 mL/min/1.73 m^2^, 23.0% with GFR 30–44 mL/min/1.73 m^2^, 20.4% with GFR 45–59, 10.9% with GFR 60–89 and 5.4% with GFR greater than or equal to 90 mL/min/1.73 m^2^. This suggests the prevalence of hypothyroidism with declining glomerular filtration rate and the authors also reported an overall prevalence of 54% for subclinical hypothyroidism [82]. In a more recent study, 43.8% subjected presented with hypothyroidism among which 41.2% had subclinical hypothyroidism and 2.7% had overt hypothyroidism [83]. The authors also found that as the severity of chronic kidney disease increases, the prevalence of hypothyroidism also rises, as evident by 3.1% of subjects with hypothyroidism in stage 3 b, 25.0% in stage 3, and 71.9% cases in stage 5 [83].

There are other observational studies that have reported prevalence of subclinical hypothyroidism among them 4.1% in a cross-sectional study of 122 chronic renal disease patients [84], 21.8% subjects in 61 patients with end stage renal disease on hemodialysis [85]. The observed hypothyroidism in chronic kidney disease patients could be due to a number of factors, including impaired iodine excretion, resulting in excess with a subsequent decreased uptake secondary to Wolff-Chaikoff effect [86] and chronic metabolic acidosis [87].

Overt hyperthyroidism was observed in 1.4% of subjects and 4.0% (6/140) of subjects with subclinical hyperthyroidism and of the latter, three cases stage 1, two had stage 2 and one had stage 4 chronic kidney disease. Overt and subclinical hyperthyroidism is not a usual finding in patients with chronic kidney disease. In a cross-sectional study, the prevalence of subclinical hyperthyroidism was 3.3% [88]. Likewise, in a multicentre cross-sectional study by Ng et al. comprising of 122 chronic kidney disease patients on peritoneal dialysis, the prevalence of subclinical hyperthyroidism was 4.1% [84]. A possible mechanism for the observed hyperthyroidism in chronic kidney disease patients is the retention of iodine in the thyroid follicle cells, owing to diminished kidney excretion through the Jod-Basedow phenomenon [88].

Our study found no difference in TSH or FT4 levels between patients who are diabetic compared to non-diabetic subjects. However, findings from other observational studies differ from our results. In a controlled study by Bando et al. comprising 32 diabetics and 31 non-diabetic subjects, the TSH level was significantly higher and serum FT4 levels significantly decreased in subjects in the diabetic group compared to those in the non-diabetic group [89]. Likewise, in a cross-sectional study of 991 subjects with type 2 diabetes mellitus, the prevalence of subclinical hypothyroidism was 12.7% and these subjects had a high risk of chronic kidney disease [90]. Notably, a number of observational studies involving diabetics and no-diabetics have indicated that subclinical hypothyroidism is related to an elevated risk of coronary heart disease events and mortality as well as cardiovascular disease risk factors [91,92].

The findings of this study with reference to the prevalence of hypothyroidism highlights the need for clinicians to perform routine screening and evaluation of thyroid function in chronic kidney disease patients. This is because the signs and symptoms of hypothyroidism are frequently disguised with the uremic state [93]. Moreover, inadequate levels of vitamin D have been associated with greater risk of cardiovascular events [94]. Future studies are recommended that will evaluate the usefulness of thyroid hormone and vitamin D supplements and their long-term properties in reducing both morbidity and mortality in chronic kidney disease patients.

## 5. Conclusions

The majority of chronic kidney disease patients had elevated serum cystatin C levels that increased in a stepwise manner with disease progression. Serum cystatin C levels were increased in patients with stage 1 and 2 disease, which suggest that this biomarker is sensitive in detecting early decline in renal function. The early detection of early renal impairment by serum cystatin C could lead to a likely targeted approach in identifying persons at greater risk of complications of chronic kidney disease.

Vitamin D deficiency and thyroid disorders are exhibited in the chronic kidney disease patients and the severity of the former rises with the progression of the disease. Chronic kidney disease patients should be routinely screened for thyroid disorders and there should be the timely determination of vitamin D levels so that, if suboptimal levels are observed, an appropriate intervention can be taken to reduce the risk of cardiovascular diseases.

## Figures and Tables

**Table 1:** Demographic and biochemical characteristics of the study population.

	Males		Females		*p*
Number	65	−46.40%	75	−53.60%	
Age range (years)	18	−94	18	–98	
Age (years)	61.11 ± 17.39		55.61 ± 17.98		0.07
Age Groups					
<29	5 (7.7%)		5 (6.7%)		
30–39	0 (0%)		9 (12.0%)		
40–49	11	−16.90%	15	−20.00%	
50–59	13	−20.00%	15	−20.00%	
60–69	14	−21.50%	14	−18.70%	
70+	22	−33.80%	17	−22.70%	
Weight range (kg)	42	–147.9	41.2–111.6		
Weight (kg)	76.21		70.3	±16.83	0.09
Height range (cm)	141		142.2	–180.3	
Height (cm)	172.23 ± 15.47		162.10 ± 7.85		<0.05
Body surface area (BSA) (m^2^)	1.87	±0.27	1.76 ± 0.21		<0.05
Serum creatinine (μmol/L)	240.86 ± 248.67		233.23 ± 247.71		0.48
Creatinine clearance (mL/min)	60.42		58.47	±59.34	0.84
Albumin (g/L)	41.48		40.04	±4.25	0.07
Calcium (mmol/L)	2.29	±0.20	2.30 ± 0.16		0.78
Vitamin D levels (ng/mL)	33.37		35	±15.33	0.54
Free Thyroxine (FT4) (ng/mL)	1.18	±0.27	1.07 ± 0.27		<0.05
TSH (mU/L)	2.25	±2.37	2.12 ± 1.53		0.68
Cystatin C (mg/L)	2.28	±1.20	2.36 ± 1.44		0.74
Urea (mmol/L)	12.03		10.88	±7.62	0.4

Data are given as mean ± standard deviation (SD). TSH—Thyroid stimulating hormone. Significant levels are given at *p* < 0.05.

**Table 2:** Biochemical characteristics of patients at normal vs. abnormal cystatin C levels.

	Cystatin C (mg/L)				
	0.61–0.95		>0.95		*p*
Study population (*n*)		11	129		-
Age (years)	36.55	±17.12	60.01	±16.73	<0.05
Creatinine (μmol/L)	51.91	±21.55	252.53	±251.36	<0.05
CrCl (mL/min)	189.00 ± 81.11		48.32	±37.71	<0.05
TSH (mU/L)	2.19	±1.40	2.19	±2.00	0.99
FT4 (ng/mL)	1.08	±0.23	1.12	±0.28	0.60
TSH (mU/L)	2.19 ± 1.40		2.19	±1.20	0.99
Albumin (g/L)	41.00 ± 4.45		40.68 ± 4.69		0.83
Urine Protein (g/day)	0.37	±0.48	0.57	±0.80	0.41

**Table 3:** Biochemical characteristics of patients at different CKD stages.

Characteristics	Stage I		Stage II		Stage III		Stage IV		Stage V		*p*
No. of Subjects		26		27		41		24		22	-
Age	44.15	±16.05	53.56	±13.10	60.59	±17.18	70.67	±14.84	62.23	±17.64	<0.05
BSA	1.87	±0.27	1.85	±0.21	1.79	±0.25	1.74	±0.27	1.80	±0.22	0.52
Creatinine (μmol/L)	76.54	±34.96	102.85 ± 39.57		170.10 ± 69.76		247.21 ± 78.46		703.36	±306.00	<0.05
Urea (mmol/L)	4.17	±1.74	6.38	±2.59	10.78 ± 5.53		15.4	±6.36	22.98 ± 7.89		<0.05
Albumin (g/L)	40.69 ± 5.73		41.00 ± 3.46		41.20 ± 5.04		39.75 ± 4.71		40.50 ± 3.89		0.32
Potassium (mmol/L)	4.28	±0.47	4.38	±0.73	4.75	±0.64	4.77	±0.66	5.10	±1.63	<0.05
Calcium (mmol/L)	2.31	±0.13	2.35	±0.12	2.34	±0.22	2.28	±0.16	2.16	±0.19	<0.05
Phosphorus (mmol/L)	1.05	±0.20	1.04	±0.19	1.10	±0.24	1.12 ± 0.21		1.41	±0.25	<0.05
Uric Acid (mmol/L)	0.50	±0.72	0.39	±0.11	0.41	±0.12	0.45	±0.13	0.41	±0.13	0.79
24-h Protein (g/day)	0.46	±0.55	0.42 ± 0.911		0.62	±0.95	0.60	±0.67	0.68	±0.63	0.72
24-h Urea (mmol/day)	292.12 ± 95.14		217.11 ± 100.11		225.38 ± 93.99		179.04 ± 59.57		128.68 ± 68.67		<0.05
FT4 (ng/mL)	1.15	±0.21	1.14	±0.33	1.17	±0.32	1.07	±0.23	1.03	±0.20	0.38
TSH (mIU/mL)	1.71	±1.00	1.94	±1.47	2.60	±2.92	2.00	±0.99	2.48	±1.82	0.45
Vitamin D (ng/mL)	33.42	±15.18	37.26	±16.49	36.00	±16.24	34.92	±15.44	27.50	±12.04	0.23
Cystatin C (mg/L)	1.11	±0.31	1.52	±0.33	2.26	±0.82	2.71 ± 0.82		4.46	±1.34	<0.05

**Table 4:** Demographics and biochemical characteristics in diabetic vs. non-diabetic patients with chronic kidney disease.

	Diabetes Mellitus		Non-Diabetic		*p*
Study population		43		97	-
Age (years)	66.00	±14.18	54.69	±18.28	<0.05
Albumin (g/L)	40.23 ± 4.09		40.92 ± 4.89		0.42
Creatinine (μmol/L)	320.42	±273.46	199.69	±226.50	<0.05
CrCl (mL/min)	34.12	±30.91	70.57	±61.97	<0.05
Cystatin C (mg/L)	2.80	±1.28	2.11	±1.30	<0.05
Urine Protein (g/day)	0.79	±0.96	0.45	±0.67	<0.05
FT4 (ng/mL)	1.10	±0.17	1.13	±0.31	0.60
TSH (mU/mL)	2.28	±1.40	2.14	±2.16	0.69
Vitamin D (ng/mL)	30.16	±14.84	36.05	±15.49	<0.05
Calcium (mmol/L)	2.28	±0.23	2.31	±0.16	0.37
Phosphorus (mmol/L)	1.20	±0.29	1.10	±0.23	<0.05

**Table 5:** Sufficient vs. not sufficient (insufficiency and deficiency) vitamin D levels compared to other variables in chronic kidney disease patients.

		Vitamin D (ng/mL)			
	<30		≥30		*p*
Study population		62		78	-
Age	56.35	±19.14	59.60	±16.76	<0.05
Albumin (g/L)	38.82 ± 5.17		42.21 ± 3.58		<0.05
Calcium (mmol/L)	2.25	±0.20	2.34	±0.16	<0.05
Phosphorus (mmol/L)	1.18	±0.25	1.09	±0.24	<0.05
Urine Protein (g/day)	0.81	±0.94	0.36	±0.56	<0.05
Creatinine (μmol/L)	307.03	±292.89	203.42	±188.34	0.10

## Data Availability

Data supporting reported results can be found at: https://figshare.com/articles/dataset/Kidney_Function_Test_Project-G2FR5-D_McGrowder_January_1_2021_xlsx/13513971; doi:10.6084/m9.figshare.13513971.
